# Supplementary material for: Sex Differences in Patent Ductus Arteriosus Incidence and Response to Pharmacological Treatment in Preterm Infants: A Systematic Review, Meta-Analysis and Meta-Regression
Source: J Pers Med. 2022 Jul 14;12(7):1143. doi: 10.3390/jpm12071143 (PMC9321725; doi:10.3390/jpm12071143)
Supplement: Supplementary file 1 [file jpm-12-01143-s001.zip › jpm-1797547-supplementary.pdf]

Supplementary Material

Sex differences in patent ductus arteriosus incidence and response to pharmacological treatment in preterm infants: A systematic review, meta-analysis and meta-regression

Table S1. Search strategy

**Pubmed**<br>(<br>(Sex[MESH] OR Sex Characteristics[MESH] OR Sex Distribution[MESH] OR "Sex Characteristic*"[tiab] OR "Gender Difference*"[tiab] OR "Sex Dimorphism*"[tiab] OR "Gender Characteristic*"[tiab] OR "Sex Difference*"[tiab] OR "Sex Distribution*"[tiab] OR "Gender Distribution*"[tiab] OR "Male Disadvantage"[tiab] OR "Female Advantage"[tiab] OR risk [tiab])<br>AND<br>(Patent ductus arteriosus[MESH] OR "Patency of the Ductus Arteriosus"[tiab] OR "Ductus Arteriosus"[tiab] OR "Ductus Botalli"[tiab] OR "PDA"[tiab])<br>)
**Embase**<br>(<br>(exp Sex Characteristics/ OR exp Sex Distribution / OR exp Sex Factors/)<br>OR ("male disadvantage" or "female advantage" or gender or sex or "gender difference" or "gender differences" or "sex difference" or "sex differences" or "gender differential" or "gender differentials" or "sex differential" or "sex differentials" or "sexual dimorphism" or "sexually dimorphic")<br>)<br>AND<br>(patent ductus arteriosus)<br>)

Table S2. Characteristics of the included studies

First author, year	Country	Prospective?	Total infants	Centers	Mean/median GA cohort	Mean/median BW cohort	Independent variable	Outcomes	PDA primary outcome	Selection	Comparability	Outcome/ Exposure	Total NOS
Adrouche-Amrani2012 (1)	USA	No	79	1	25.8	770.7	PDA	hsPDA / Treatment	YES	3	1	3	**7**
Ahamed 2015 (2)	USA	No	203	1	26.5	840.6	PDA	hsPDA / Treatment	NO	3	1	3	**7**
Ahmed 2020 (3)	Egypt	Yes	75	1	31.7	ND	PDA	Any PDA	YES	2	1	3	**6**
Aikio 2014 (4)	Finland	No	88	1	28.6	1308.0	PDA	hsPDA	YES	3	1	3	**7**
Akar 2019 (5)	Turkey	No	389	1	29.5	1054.8	PDA	hsPDA	YES	3	2	2	**7**
Alan 2013 (6)	Turkey	Yes	25	1	27.8	988.1	PDA	hsPDA	YES	3	1	3	**7**
Antonucci 2009 (7)	Italy	Yes	40	1	29.5	1305.0	PDA	hsPDA	NO	2	2	3	**7**
Asrani 2018 (8)	USA	Yes	70	1	27.9	1014.3	PDA	hsPDA	YES	3	2	3	**8**
Bas 2014 (9)	Spain	No	194	2	27.9	1008.5	PDA	hsPDA / Treatment	YES	3	1	3	**7**
Becerra 2013 (10)	Mexico	Yes	66	1	28.0	950.0	PDA	hsPDA	YES	3	1	3	**7**
Bekmez 2018 (11)	Turkey	No	212	1	29.0	1237.8	PDA	hsPDA	YES	3	2	3	**8**
Bertino 2009 (12)	Italy	Yes	262	1	30.4	1140.0	Sex	hsPDA	NO	3	2	2	**7**
Boghossian 2018 (13)	USA	No	194112	852	26.0	919	Sex	hsPDA	NO	4	2	3	**9**
Bonamy 2017 (14)	11 European countries	No	6896	Multi	29.1	1223.0	PDA	hsPDA	NO	4	1	3	**8**
Boo 2006 (15)	Malaysia	Yes	60	1	28.3	1115.8	PDA	Treatment	NO	3	2	3	**8**
Bravo 2011 (16)	Spain	Yes	90	1	28.4	1066.7	PDA	Treatment	YES	4	2	3	**9**
Breatnach 2017a (17)	Ireland	No	121	1	26.8	968.0	PDA	Any PDA	NO	3	2	3	**8**
Breatnach 2017b (18)	Ireland	No	51	1	27	1060	PDA	Any PDA	NO	3	1	3	**7**
Brooks 2005 (19)	Australia	No	252	1	26.0	900.5	PDA	Treatment	YES	3	2	3	**8**
Brunner 2013 (20)	Austria	Yes	322	1	28.5	1174.0	PDA	Any PDA	NO	3	1	3	**7**

First author, year	Country	Prospective?	Total infants	Centers	Mean/median GA cohort	Mean/median BW cohort	Independent variable	Outcomes	PDA primary outcome	Selection	Comparability	Outcome/ Exposure	Total NOS
Buddhe 2012 (21)	USA	Yes	69	2	27.6	981.0	PDA	hsPDA	YES	3	1	3	**7**
Cakir 2019 (22)	Turkey	No	799	1	28.0	1068.2	PDA	hsPDA	YES	4	2	3	**9**
Chen 2015 (23)	China	Yes	77	1	29.5	1152.7	PDA	Any PDA / Treatment	NO	3	1	3	**7**
Chessex 2010 (24)	Canada	Yes	40	1	25.4	752.0	Sex	hsPDA	NO	2	2	3	**7**
Chock 2011 (25)	USA	Yes	31	2	26.2	850.9	PDA	hsPDA / Any PDA /Treatment	NO	2	2	3	**7**
Chock 2014 (26)	USA	No	187	1	27.6	1004.9	PDA	hsPDA	NO	3	1	3	**7**
Chock 2016 (27)	USA	Yes	47	1	27.2	865.4	PDA	hsPDA / Any PDA	NO	3	2	3	**8**
Choi 2005 (28)	Korea	Yes	66	1	29.9	1356.0	PDA	hsPDA	YES	3	2	3	**8**
Clyman 2020 (29)	North America	No	407	1	26.2	831.3	PDA	hsPDA	NO	3	1	3	**7**
Collins 2018 (30)	USA	Yes	1090	8	33.0	1410.8	PDA	hsPDA / Any PDA	NO	4	1	3	**8**
Coviello 2020 (31)	Italy	Yes	60	1	28.8	1223.2	PDA	hsPDA	YES	3	2	3	**8**
Dagle 2009 (32)	USA	No	366	1	27.9	1042.9	PDA	Any PDA	NO	3	2	3	**8**
Dagle 2019 (33)	USA	No	1013	Multi	25.9	762.7	PDA	hsPDA	YES	4	1	3	**8**
Dani 2008 (34)	Italy	Yes	34	1	25.6	786.7	PDA	hsPDA /Any PDA /Treatment	YES	4	2	3	**9**
Demir 2016 (35)	Turkey	No	235	1	29.0	1548.5	PDA	hsPDA	YES	4	1	3	**8**
Derzbach 2005 (36)	Hungary	No	140	1	29.0	1189.8	Sex	hsPDA	NO	3	2	3	**8**
Dix 2016 (37)	The Netherlands	Yes	398	1	29.1	1312.4	PDA	hsPDA /Any PDA /Treatment	YES	3	1	3	**7**
El-Khuffash 2008 (38)	Ireland	Yes	80	1	28.0	1060.0	PDA	hsPDA /Any PDA	NO	3	2	3	**8**
Elsmen 2004 (39)	Sweden	No	175	1	26.0	771.0	Sex	hsPDA	NO	3	1	3	**7**
Engeseth 2020 (40)	Germany	Yes	228	1	26.7	865.4	PDA	Any PDA / Treatment	NO	3	1	3	**7**
Engür 2015 (41)	Turkey	No	63	1	29.8	1330.8	PDA	hsPDA/ Any PDA	YES	3	2	3	**8**

First author, year	Country	Prospective?	Total infants	Centers	Mean/median GA cohort	Mean/median BW cohort	Independent variable	Outcomes	PDA primary outcome	Selection	Comparability	Outcome/ Exposure	Total NOS
Fink 2018 (42)	Israel	No	72	1	27.3	1016.4	PDA	hsPDA	YES	3	2	3	**8**
Flood 2015 (43)	USA	No	221	1	ND	1680.0	PDA	Any PDA	YES	3	2	3	**8**
Furzan 1985 (44)	USA	Yes	120	1	29.4	1165.0	PDA	hsPDA	YES	4	2	3	**9**
Ghirardello 2020 (45)	Italy	Yes	151	1	29.1	1098.4	PDA	hsPDA	YES	3	1	3	**7**
Godambe 2006 (46)	Canada	No	107	1	26.3	887.4	PDA	Treatment	YES	3	2	3	**8**
Gomez-Pomar 2017 (47)	USA	Yes	31	1	26.0	846.7	PDA	Any PDA	NO	2	2	3	**7**
Goudjil 2013 (48)	France	Yes	44	1	28.5	1209.5	PDA	hsPDA	NO	3	1	3	**7**
Griesmaier 2014 (49)	Austria	No	156	1	30.5	1393.9	PDA	Any PDA	NO	3	1	3	**7**
Grosfeld 1996 (50)	USA	Yes	1016	1	28.4	1053.8	PDA	hsPDA/ Treatment	NO	4	1	3	**8**
Halil 2018 (51)	Turkey	No	162	1	27.7	986.2	PDA	Treatment	YES	3	1	3	**7**
Halliday 1979 (52)	USA	Yes	36	1	27.9	906.7	PDA	Treatment	Yes	3	1	3	**7**
Hammoud 2003 (53)	Kuwait	No	101	1	29.9	1094.4	PDA	hsPDA	YES	3	1	3	**7**
Härkin 2016 (54)	Finland	No	23	1	28.4	1150	PDA	Treatment	YES	3	1	3	**7**
Härkin 2018 (55)	Finland	No	3668	Multi	29.1	1243.0	PDA	hsPDA / Treatment	YES	4	1	3	**8**
Harris 2018 (56)	New Zealand	No	51	1	27.8	1093.8	PDA	hsPDA	NO	3	2	3	**8**
Harris 2020 (57)	UK	Yes	318	Multi	26.9	894.8	Sex	Any PDA	NO	3	1	4	**8**
Hintz 2006 (58)	USA	No	2553	1	25.3	767.1	Sex	Any PDA	NO	4	1	3	**8**
Hoodbhoy 2009 (59)	UK	Yes	44	1	25.7	763.9	PDA	Any PDA	NO	3	2	3	**8**
Hsu 2010 (60)	Taiwan	Yes	31	1	30.0	1396.0	PDA	Treatment	NO	2	2	3	**7**
Hsu 2019 (61)	Taiwan	No	18	1	27.6	1075.0	PDA	Treatment	YES	3	1	3	**7**
Inayat 2015 (62)	USA	Yes	53	1	26.6	971.9	PDA	hsPDA	YES	4	2	3	**9**
Isayama 2015 (63)	Japan,	No	6183	Multi	27.9	1052.2	PDA	hsPDA	NO	4	2	3	**9**

First author, year	Country	Prospective?	Total infants	Centers	Mean/median GA cohort	Mean/median BW cohort	Independent variable	Outcomes	PDA primary outcome	Selection	Comparability	Outcome/ Exposure	Total NOS
	Canada												
Ito 2017 (64)	Japan	No	38023	Multi	28.0	1034	Sex	hsPDA	NO	4	1	3	**8**
Janz-Robinson 2015 (65)	Australia	No	1473	10	26.5	945.0	PDA	hsPDA / Treatment	NO	4	1	3	**8**
Jennische 2003 (66)	Sweden	No	64	1	28.9	1306	Sex	hsPDA	NO	3	1	3	**7**
Jhaveri 2010 (67)	USA	No	396	1	25.8	828.6	PDA	Treatment	YES	3	1	3	**7**
Jim 2005 (68)	China	Yes	77	1	28.2	1087.2	PDA	hsPDA	NO	4	2	3	**9**
Jones 2005 (69)	Canada	Yes	3117	17	27.4	1071	Sex	hsPDA	NO	4	2	3	**9**
Juujärvi 2018 (70)	Finland	No	295	1	26.4	821.7	PDA	Treatment	YES	3	1	3	**7**
Kahvecioglu 2018 (71)	Turkey	Yes	60	1	27.8	1024.6	PDA	Any PDA	YES	3	1	3	**7**
Kara 2019 (72)	Turkey	Yes	60	1	29.0	1069.2	PDA	hsPDA	YES	3	1	3	**7**
Karabulut 2019 (73)	Turkey	No	148	2	28.8	1092.0	PDA	hsPDA	YES	3	2	3	**8**
Katheria 2018 (74)	USA	No	292	1	28.4	1202.1	PDA	hsPDA	YES	3	2	3	**8**
Kaur 2019 (75)	Canada	No	183	1	26.4	870.0	PDA	Treatment	YES	3	1	3	**7**
Kazanci 2019 (76)	Turkey	No	481	1	28.0	1067.7	PDA	hsPDA	YES	4	2	3	**9**
Kent 2012 (77)	Australia	No	2549	1	26.3	923.0	Sex	hsPDA	NO	4	1	3	**8**
Kim 2010 (78)	Korea	Yes	78	1	26.5	742.0	PDA	Treatment	YES	2	2	3	**7**
Kim 2012 (79)	Korea	Yes	28	1	32.7	1918.0	PDA	hsPDA	YES	2	2	3	**7**
Kwinta 2009 (80)	Poland	Yes	60	1	28.5	1087.0	PDA	hsPDA	YES	4	2	3	**9**
Laughon2007 (81)	USA	No	28413	220	28.2	1089.7	PDA	hsPDA	YES	3	2	3	**8**
Lauterbach 2001 (82)	USA	No	51	1	32.3	2009.9	Sex	hsPDA	NO	2	1	3	**6**
Ledo 2017 (83)	Spain	Yes	72	1	28.1	1154.6	PDA	hsPDA /Any PDA	NO	3	1	3	**7**
Leonhardt 1987 (84)	Germany	Yes	32	1	30.0	1095.0	PDA	Treatment	YES	3	2	3	**8**
Letshwiti 2014 (85)	Ireland	Yes	69	1	28.6	1119.0	PDA	hsPDA	YES	4	2	3	**9**

First author, year	Country	Prospective?	Total infants	Centers	Mean/median GA cohort	Mean/median BW cohort	Independent variable	Outcomes	PDA primary outcome	Selection	Comparability	Outcome/ Exposure	Total NOS
Li 2013 (86)	Multiple country	No	289	1	27.7	1089.0	PDA	hsPDA	NO	2	2	3	**7**
Louis 2018 (87)	Canada	No	98	1	25.4	772.0	PDA	Treatment	YES	3	1	3	**7**
Madan 2008 (88)	USA	No	210	1	26.3	914.7	PDA	Treatment	YES	3	2	3	**8**
Madan 2009 (89)	USA	Yes	2300	Multi	25.2	736.2	PDA	Treatment	NO	4	2	2	**8**
Martin 1982 (90)	USA	Yes	20	1	31.2	1629.5	PDA	hsPDA /Any PDA	NO	3	1	3	**7**
Meinarde 2016 (91)	Argentina	No	88	1	28.4	1112.0	PDA	hsPDA	YES	3	1	3	**7**
Mellander 1984 (92)	USA	Yes	42	1	27.8	830.0	PDA	Treatment	YES	3	2	3	**8**
Mine 2013 (93)	Japan	Yes	46	1	28.1	954.4	PDA	hsPDA	YES	4	2	3	**9**
Natarajan 2013 (94)	USA	No	968	Multi	25.8	761.5	PDA	hsPDA	NO	4	2	3	**9**
Nemerofsky 2008 (95)	USA	Yes	125	1	27.5	1019.0	PDA	hsPDA	YES	4	2	3	**9**
Neubauer 2012 (96)	Austria	Yes	408	1	29.3	1259.2	Sex	hsPDA	NO	4	1	3	**8**
Nizarali 2012 (97)	Portugal	No	318	1	29.0	1139.2	PDA	hsPDA	NO	3	2	3	**8**
Nuntnarumit 2009 (98)	Thailand	Yes	35	1	30.3	1322.3	PDA	hsPDA	YES	2	2	3	**7**
Oh 2020 (99)	Korea	No	92	1	27.0	939.9	PDA	Treatment	YES	3	2	3	**8**
Okur 2019 (100)	Turkey	Yes	119	1	28.6	1068.6	PDA	hsPDA	YES	3	1	3	**7**
Oliveira 2016 (101)	Portugal	No	328	1	30.0	1231.0	PDA	Any PDA	YES	4	1	3	**8**
Olsson 2018 (102)	Sweden	Yes	47	1	25.2	738.3	PDA	hsPDA	YES	3	1	3	**7**
Olsson 2019 (103)	Sweden	Yes	60	1	25.3	732.3	PDA	hsPDA	YES	3	1	3	**7**
Olukman 2017 (104)	Turkey	No	824	1	29.6	1193.1	PDA	hsPDA	YES	4	2	3	**9**
O´Rourke 2008 (105)	Ireland	No	95	1	28.8	1000.0	PDA	hsPDA	NO	3	1	3	**7**
Patole 2007 (106)	Australia	No	252	1	26.0	900.5	PDA	hsPDA /Any PDA	NO	4	2	3	**9**
Peacock 2012 (107)	United Kingdom	Yes	797	Multi	26.5	853.0	Sex	Any PDA	NO	4	1	2	**7**

First author, year	Country	Prospective?	Total infants	Centers	Mean/median GA cohort	Mean/median BW cohort	Independent variable	Outcomes	PDA primary outcome	Selection	Comparability	Outcome/ Exposure	Total NOS
Pees 2010 (108)	Germany	Yes	15	1	26.2	853.8	PDA	hsPDA / Treatment	YES	2	2	3	**7**
Pereira 2018 (109)	UK	No	59	1	25.8	817.0	PDA	hsPDA / Treatment	YES	3	2	3	**8**
Polat 2016 (110)	Turkey	Yes	58	1	26.6	824.1	PDA	hsPDA	YES	2	2	3	**7**
Pourarian 2017 (111)	Iran	Yes	200	1	32.1	1741.0	PDA	hsPDA /Any PDA	YES	3	1	3	**7**
Reller 1985 (112)	USA	Yes	50	1	29.2	1166.4	PDA	hsPDA	YES	3	1	3	**7**
Rodriguez 2018 (113)	Spain	Yes	85	1	29.8	1222.6	PDA	hsPDA	YES	4	2	3	**9**
Romagnoli 2018 (114)	USA	Yes	65	1	27.5	1019.0	BW	Any PDA	YES	2	1	3	**6**
Rooney 2019 (115)	USA	Yes	144	1	26.0	ND	PDA	Treatment	YES	2	2	3	**7**
Sadeck 2014 (116)	Brazil	No	494	16	27.3	787.1	PDA	hsPDA / Treatment	YES	3	1	3	**7**
Sallmon 2012 (117)	Germany	No	1350	2	28.3	1060.0	PDA	hsPDA /Any PDA /Treatment	YES	4	2	3	**9**
Sallmon 2018 (118)	Germany	No	471	2	26.0	867.0	PDA	Treatment	YES	3	2	3	**8**
Sanjeev 2005 (119)	USA	Yes	29	2	26.3	873.5	PDA	hsPDA	YES	3	2	2	**7**
Schwarz 2018 (120)	Germany	Yes	22	1	28.6	1076.0	PDA	hsPDA	YES	3	1	3	**7**
Sellmer 2013 (121)	Denmark	Yes	183	1	28.1	1066.1	PDA	hsPDA/ Any PDA	NO	3	1	3	**7**
Semberova 2017 (122)	Ireland	No	280	3	28.9	1126.3	PDA	Any PDA	YES	3	2	3	**8**
Seon 2013 (123)	Korea	No	58	1	27.1	946.0	PDA	Treatment	NO	3	1	2	**6**
Shah 2011 (124)	USA	Yes	497	1	25.8	825.1	PDA	Any PDA /Treatment	YES	3	2	3	**8**
Skiold 2014 (125)	Sweden	Yes	107	1	25.6	800.6	Sex	hsPDA	NO	3	1	3	**7**
Stark 2011 (126)	Australia	Yes	60	1	30.0	1287.5	BW	hsPDA	NO	3	1	3	**7**
Steiner 2015 (127)	Austria	No	34	1	26.0	807.0	Sex	Treatment	YES	2	2	3	**7**
Stevenson 2000 (128)	USA	No	6738	12	28,5	~1100	Sex	hsPDA	NO	4	1	3	**8**
Strauss 2011 (129)	Israel	No	120	1	27.9	1019.0	PDA	hsPDA	YES	3	2	3	**8**

First author, year	Country	Prospective?	Total infants	Centers	Mean/median GA cohort	Mean/median BW cohort	Independent variable	Outcomes	PDA primary outcome	Selection	Comparability	Outcome/ Exposure	Total NOS
Tauber 2016 (130)	USA	Yes	95	1	28.5	1185.2	PDA	hsPDA/ Any PDA	NO	3	2	3	**8**
Terek 2014 (131)	Turkey	No	292	1	29.7	1470.2	PDA	hsPDA/ Any PDA	YES	3	1	3	**7**
Tioseco 2006 (132)	USA	No	833	1	30.9	1546.8	Sex	hsPDA	NO	4	2	2	**8**
Uchiyama 2011 (133)	Japan	No	57	1	28.3	1040.6	PDA	Treatment	NO	2	2	3	**7**
Vaidya 2021 (134)	USA	No	66	1	25.5	783	BW	Treatment	YES	3	1	3	**7**
Van de Bor 1988 (135)	Netherlands	No	1252	Multi	30.5	1269.7	PDA	hsPDA	NO	4	2	3	**9**
Velazquez 2018 (136)	USA	No	151	1	26.0	800.3	PDA	hsPDA	NO	3	1	3	**7**
Vieux 2010 (137)	France	No	148	3	28.3	1114.3	PDA	hsPDA	NO	3	1	3	**7**
Visconti 2013 (138)	Brazil	No	67	1	28.2	1063.3	PDA	hsPDA	YES	4	2	3	**9**
Weiss 1995 (139)	USA	Yes	77	1	27.8	1076.6	PDA	Treatment	YES	3	1	3	**7**
Weisz 2017 (140)	Canada	No	754	3	25.8	812.3	PDA	Treatment	YES	4	1	3	**8**
Yang 2008 (141)	Singapore	No	139	1	26.3	807.3	PDA	Treatment	NO	3	1	3	**7**
Yoon 2007 (142)	Korea	No	29	3	31.4	1651.2	PDA	Treatment	YES	3	1	3	**7**
Yum 2018 (143)	Korea	Yes	71	1	27.9	1038.3	PDA	hsPDA	YES	3	2	3	**8**
Zanardo 2008 (144)	Italy	Yes	46	1	27.5	1031.3	PDA	Treatment	YES	3	1	3	**7**
Zisk 2011 (145)	USA	No	12807	Multi	29.4	1358.6	Sex	hsPDA/ Any PDA	NO	4	2	3	**9**
Zozaya 2019 (146)	Spain	No	ND	Multi	ND	ND	Sex	hsPDA	NO	4	1	3	**8**

hs: hemodynamically significant; NOS: Newcastle-Ottawa Scale; PDA: patent ductus arteriosus

1. Adrouche-Amrani, L.; Green, R.S.; Gluck, K.M.; Lin, J. Failure of a repeat course of cyclooxygenase inhibitor to close a PDA is a risk factor for developing chronic lung disease in ELBW infants. *BMC Pediatr.* **2012**, *12*, 10. https://doi.org/10.1186/1471-2431-12-10.

2. Ahamed, M.; Verma, P.; Lee, S.; Vega, M.; Wang, D.; Kim, M.; Fuloria, M. Predictors of successful closure of patent ductus arteriosus with indomethacin. *J. Perinatol.* **2015**, *35*, 729. https://doi.org/10.1038/jp.2015.33.
3. Ahmed, E.G.; Samra, N.M.; Amin, S.A.; Borayek, H.A.; Abdelrazek, G. Platelets and platelet derived growth factor and ductus arteriosus in preterm neonates. *Progress in Pediatr. Cardiol.* **2020**, 101226. https://doi.org/10.1016/j.ppedcard.2020.101226.
4. Aikio, O.; Harkin, P.; Saarela, T.; Hallman, M. Early paracetamol treatment associated with lowered risk of persistent ductus arteriosus in very preterm infants. *J Matern Fetal Neonatal Med.* **2014**, *27*, 1252-1256. https://doi.org/10.3109/14767058.2013.854327
5. Akar, S.; Topcuoglu, S.; Tuten, A.; Ozalkaya, E.; Karatepe, H.O.; Gokmen, T.; Ovali, F.; Karatekin, G. Is the First Postnatal Platelet Mass as an Indicator of Patent Ductus Arteriosus? *Arch. Iran. Med.* **2019**, *22*, 687.
6. Alan, S.; Karadeniz, C.; Okulu, E.; Kılıc, A.; Erdeve, O.; Ucar, T.; Atasay, B.; Atalay, S.; Arsan, S. Management of patent ductus arteriosus in preterm infants: clinical judgment might be a fair option. *J. Matern.-Fetal Neonatal Med.* **2013**, *26*, 1850-1854. https://doi.org/10.3109/14767058.2013.801956.
7. Antonucci, R.; Cuzzolin, L.; Arceri, A.; Dessì, A.; Fanos, V. Changes in urinary PGE 2 after ibuprofen treatment in preterm infants with patent ductus arteriosus. *Eur. J. Clin. Pharmacol.* **2009**, *65*, 223-230. https://doi.org/10.1007/s00228-008-0586-3.
8. Asrani, P.; Aly, A. M.; Jiwani, A. K; Niebuhr; B. R; Christenson, R.H.; Jain, S.K.; High-sensitivity troponin T in preterm infants with a hemodynamically significant patent ductus arteriosus. *J. Perinatol.* **2018** *38(11),* 1483-1489. https://doi.org/10.1038/s41372-018-0192-x
9. Bas-Suárez, M.P.; González-Luis, G.E.; Saavedra, P.; Villamor, E. Platelet counts in the first seven days of life and patent ductus arteriosus in preterm very low-birth-weight infants. *Neonatology* **2014**, *106*, 188-194. https://doi.org/10.1159/000362432.
10. Becerra, G.H.; Bernárdez Zapata, I.; Iglesias Leboreiro, J.; Bahena, E.J.P.; Rendón Macías, M.E. Medical care of children‹ 30 weeks gestation with ductus arteriosus persistent. *Rev. Mex. de Pediatria* **2013**, *80*, 131-135.
11. Bekmez, B. Ö.; Tayman, C.; Büyüktiryaki, M.; Çetinkaya, A K; Çakır, U.; Derme, T. A promising, novel index in the diagnosis and follow-up of patent ductus arteriosus: Red cell distribution width-to-platelet ratio. *J. Clin. Lab. Anal*. **2018** *32(9):e22616*. https://doi.org/10.1002/jcla.22616.
12. Bertino, E.; Coscia, A.; Boni, L.; Rossi, C.; Martano, C.; Giuliani, F.; Fabris, C.; Spada, E.; Zolin, A.; Milani, S. Weight growth velocity of very low birth weight infants: role of gender, gestational age and major morbidities. *Early Hum. Dev.* **2009**, *85*, 339-347. https://doi.org/10.1016/j.earlhumdev.2008.12.014.
13. Boghossian, N.S.; Geraci, M.; Edwards, E.M.; Horbar, J.D. Sex differences in mortality and morbidity of infants born at less than 30 weeks' gestation. *Pediatrics.* **2018**, *142*. https://doi.org/10.1542/peds.2018-2352.
14. Bonamy, A.-K.E.; Gudmundsdottir, A.; Maier, R.F.; Toome, L.; Zeitlin, J.; Bonet, M.; Fenton, A.; Hasselager, A.B.; Van Heijst, A.; Gortner, L. Patent ductus arteriosus treatment in very preterm infants: a European population-based cohort study (EPICE) on variation and outcomes. *Neonatology* **2017**, *111*, 367-375. https://doi.org/10.1159/000454798.
15. Boo, N.Y.; Mohd-Amin, I.; Bilkis, A.; Yong-Junina, F. Predictors of failed closure of patent ductus arteriosus with indomethacin. *Singapore Med J* **2006**, *47*, 763. https://doi.org/10.1080/02841850600731415
16. Bravo Laguna, M.C. Evaluación del tratamiento farmacológico convencional para el cierre del ductus arterioso persistente en el recién nacido pretérmino: impacto de nuevas líneas terapéuticas. **2011**. https://repositorio.uam.es/bitstream/handle/10486/7350/41683_bravo_laguna_mari_carmen.pdf?sequence=1
17. Breatnach, C.R.; Franklin, O.; James, A.T.; McCallion, N.; Afif, E.-K. The impact of a hyperdynamic left ventricle on right ventricular function measurements in preterm infants with a patent ductus arteriosus. *Arch. Dis. Child. Fetal Neonatal Ed.* **2017**, *102*, F446-F450. https://doi.org/10.1136/archdischild-2016-311189.

18. Breatnach, C.R.; Franklin, O.; McCallion, N.; Afif, E.-K. The effect of a significant patent ductus arteriosus on Doppler flow patterns of preductal vessels: an assessment of the brachiocephalic artery. *J Pediatr.* **2017**, *180*, 279-281. e271. https://doi.org/10.1016/j.jpeds.2016.09.029.
19. Brooks, J.; Travadi, J.; Patole, S.; Doherty, D.; Simmer, K. Is surgical ligation of patent ductus arteriosus necessary? The Western Australian experience of conservative management. *Arch. Dis. Child. Fetal Neonatal Ed.* **2005**, *90*, F235-FF239. https://doi.org/10.1136/adc.2004.057638.
20. Brunner, B.; Hoeck, M.; Schermer, E.; Streif, W.; Kiechl-Kohlendorfer, U. Patent ductus arteriosus, low platelets, cyclooxygenase inhibitors, and intraventricular hemorrhage in very low birth weight preterm infants. *J. Pediatr.* **2013**, *163*, 23-28. https://doi.org/10.1071/PVv2013n163other
21. Buddhe, S.; Dhuper, S.; Kim, R.; Weichbrod, L.; Mahdi, E.; Shah, N.; Kona, S.; Sokal, M. NT-proBNP levels improve the ability of predicting a hemodynamically significant patent ductus arteriosus in very low-birth-weight infants. *J. Clin. Neonatol.* **2012**, *1*, 82. https://doi.org/10.4103/2249-4847.96758.
22. Cakir, U.; Tayman, C. A mystery of patent ductus arteriosus and serum osmolality in preterm infants. *American J. Perinatol.* **2019**, *36*, 641-646. https://doi.org/10.1055/s-0038-1673397.
23. Chen, H.-L.; Yang, R.-C.; Lee, W.-T.; Lee, P.-L.; Hsu, J.-H.; Wu, J.-R.; Dai, Z.-K. Lung function in very preterm infants with patent ductus arteriosus under conservative management: an observational study. *BMC Pediatr.* **2015**, *15*, 1-6. https://doi.org/10.1186/s12887-015-0480-y.
24. Chessex, P.; Khashu, M.; Harrison, A.; Hosking, M.; Sargent, M.; Lavoie, J.-C. Early life events, sex, and arterial blood pressure in critically ill infants. *Pediatr. Crit. Care Med.* **2010**, *11*, 75-81. https://doi.org/10.1097/PCC.0b013e3181b010f8.
25. Chock, V.Y.; Punn, R.; Oza, A.; Benitz, W.E.; Van Meurs, K.P.; Whittemore, A.S.; Behzadian, F.; Silverman, N.H. Predictors of bronchopulmonary dysplasia or death in premature infants with a patent ductus arteriosus. *Pediatr. Res.* **2014**, *75*, 570. https://doi.org/10.1038/pr.2013.253.
26. Chock, V.Y.; Ramamoorthy, C.; Van Meurs, K.P. Cerebral oxygenation during different treatment strategies for a patent ductus arteriosus. *Neonatology* **2011**, *100*, 233-240. https://doi.org/10.1159/000325149.
27. Chock, V.Y.; Rose, L.A.; Mante, J.V.; Punn, R. Near-infrared spectroscopy for detection of a significant patent ductus arteriosus. *Pediatr. Res.* **2016**, *80*, 675. https://doi.org/10.1038/pr.2016.148.
28. Choi, B.M.; Lee, K.H.; Eun, B.L.; Yoo, K.H.; Hong, Y.S.; Son, C.S.; Lee, J.W. Utility of rapid B-type natriuretic peptide assay for diagnosis of symptomatic patent ductus arteriosus in preterm infants. *Pediatrics-English Edition* **2005**, *115*, e255. https://doi.org/10.1542/peds.2004-1837.
29. Clyman, R.I.; Hills, N.K. The effect of prolonged tracheal intubation on the association between patent ductus arteriosus and bronchopulmonary dysplasia (grades 2 and 3). *J. Perinatol.* **2020**, *40*, 1358-1365. https://doi.org/10.1038/s41372-020-0718-x.
30. Collins, R.T., 2nd; Lyle, R.E.; Rettiganti, M.; Gossett, J.M.; Robbins, J.M.; Casey, P.H. Long-Term Neurodevelopment of Low-Birthweight, Preterm Infants with Patent Ductus Arteriosus. *J. Pediatr.* **2018**, *203*, 170-176 e171. https://doi.org/10.1016/j.jpeds.2018.08.004
31. Coviello, C.; Tataranno, M.L.; Corsini, I.; Leonardi, V.; Longini, M.; Bazzini, F.; Buonocore, G.; Dani, C. Isoprostanes as biomarker for patent ductus arteriosus in preterm infants. *Front. Pediatr.* **2020**, *8*, 555. https://doi.org/10.3389/fped.2020.00555.
32. Dagle, J.M.; Lepp, N.T.; Cooper, M.E.; Schaa, K.L.; Kelsey, K.J.; Orr, K.L.; Caprau, D.; Zimmerman, C.R.; Steffen, K.M.; Johnson, K.J. Determination of genetic predisposition to patent ductus arteriosus in preterm infants. *Pediatrics* **2009**, *123*, 1116. https://doi.org/10.1542/peds.2008-0313.
63. Dagle, J.M.; Ryckman, K.K.; Spracklen, C.N.; Momany, A.M.; Cotten, C.M.; Levy, J.; Page, G.P.; Bell, E.F.; Carlo, W.A.; Shankaran, S. Genetic variants associated with patent ductus arteriosus in extremely preterm infants. *J. Perinatol.* **2019**, *39*, 401. https://doi.org/10.1038/s41372-018-0285-6.
34. Dani, C.; Bertini, G.; Corsini, I.; Elia, S.; Vangi, V.; Pratesi, S.; Rubaltelli, F.F. The fate of ductus arteriosus in infants at 23-27 weeks of gestation: from spontaneous closure to ibuprofen resistance. *Acta Paediatr.* **2008**, *97*, 1176-1180. https://doi.org/10.1111/j.1651-2227.2008.00871.x

35. Demir, N.; Peker, E.; Ece, İ.; Ağengin, K.; Bulan, K.A.; Tuncer, O. Is platelet mass a more significant indicator than platelet count of closure of patent ductus arteriosus? *J. Matern.-Fetal Neonatal Med.* **2016**, *29*, 1915-1918. https://doi.org/10.3109/14767058.2015.1067296.
36. Derzbach, L.; Treszl, A.; Balogh, Á.; Vásárhelyi, B.; Tulassay, T.; Rigó J, J. Gender dependent association between perinatal morbidity and estrogen receptor-alpha Pvull polymorphism. *J. Perinat Med*. **2005**; *33(5):*461-2. https://doi.org/10.1515/JPM.2005.082.
37. Dix, L.; Molenschot, M.; Breur, J.; de Vries, W.; Vijlbrief, D.; Groenendaal, F.; Van Bel, F.; Lemmers, P. Cerebral oxygenation and echocardiographic parameters in preterm neonates with a patent ductus arteriosus: an observational study. *Arch. Dis. Child. Fetal Neonatal Ed.* **2016**, *101*, F520-F526. https://doi.org/10.1136/archdischild-2015-309192.
38. El-Khuffash, A.F.; Molloy, E.J. Influence of a patent ductus arteriosus on cardiac troponin T levels in preterm infants. *J. Pediatr.* **2008**, *153*, 350-353. https://doi.org/10.1016/j.jpeds.2008.04.014.
39. Elsmen, E.; Hansen Pupp, I.; Hellstrom-Westas, L. Preterm male infants need more initial respiratory and circulatory support than female infants. *Acta Paediatr* **2004**, *93*, 529-533. https://doi.org/10.1080/08035250410024998.
40. Engeseth, M.S.; Engan, M.; Clemm, H.; Vollsæter, M.; Nilsen, R.M.; Markestad, T.; Halvorsen, T.; Røksund, O.D. Voice and Exercise Related Respiratory Symptoms in Extremely Preterm Born Children After Neonatal Patent Ductus Arteriosus. *Front. Pediatr.* **2020**, *8*, 150. https://doi.org/10.3389/fped.2020.00150.
41. Engür, D.; Kaynak-Türkmen, M.; Deveci, M.; Yenisey, Ç. Platelets and platelet-derived growth factor in closure of the ductus arteriosus. *Turk J. Pediatr.* **2015**, *57*, 242-247. https://doi.org/10.1787/qna-v2015-1-32-fr
42. Fink, D.; Nitzan, I.; Bin-Nun, A.; Mimouni, F.; Hammerman, C. Ductus arteriosus outcome with focus on the initially patent but hemodynamically insignificant ductus in preterm neonates. *J. Perinatol.* **2018**, *38*, 1526. https://doi.org/10.1038/s41372-018-0204-x.
43. Flood, T.; Guthrie, J.D. Echocardiographic markers for the prediction of nonclosure of the patent ductus arteriosus in premature neonates. *J. Diagn. Med. Sonogr.* **2015**, *31*, 22-27. https://doi.org/10.1177/8756479314563538.
44. Furzan, J.A.; Reisch, J.; Tyson, J.E.; Laird, P.; Rosenfeld, C.R. Incidence and risk factors for symptomatic patent ductus arteriosus among inborn very-low-birth-weight infants. *Early Hum. Dev.* **1985**, *12*, 39-48. https://doi.org/10.1016/0378-3782(85)90135-5.
45. Ghirardello, S.; Raffaeli, G.; Crippa, B.L.; Gulden, S.; Amodeo, I.; Consonni, D.; Cavallaro, G.; Schena, F.; Mosca, F. The Thromboelastographic Profile at Birth in Very Preterm Newborns with Patent Ductus Arteriosus. *Neonatology* **2020**, *117 (3*), 316-323. https://doi.org/10.1159/000507553.
46. Godambe, S.; Newby, B.; Shah, V.; Shah, P.S. Effect of indomethacin on closure of ductus arteriosus in very-low-birthweight neonates 1. *Acta Pædiatrica* **2006**, *95*, 1389-1393. https://doi.org/10.1080/08035250600615150.
47. Gomez-Pomar, E.; Makhoul, M.; Westgate, P.M.; Ibonia, K.T.; Patwardhan, A.; Giannone, P.J.; Bada, H.S.; Jawdeh, E.G.A. Relationship between perfusion index and patent ductus arteriosus in preterm infants. *Pediatr. Res.* **2017**, *81*, 775. https://doi.org/10.1038/pr.2017.10.
48. Goudjil, S.; Imestouren, F.; Chazal, C.; Ghostine, G.; Wallois, F.; Leke, A.; Kongolo, G. Patent ductus arteriosus in preterm infants is associated with cardiac autonomic alteration and predominant parasympathetic stimulation. *Early Hum. Dev.* **2013**, *89*, 631-634. https://doi.org/10.1016/j.earlhumdev.2013.04.008.
49. Griesmaier, E.; Santuari, E.; Edlinger, M.; Neubauer, V.; Waltner-Romen, M.; Kiechl-Kohlendorfer, U. Differences in the maturation of amplitude-integrated EEG signals in male and female preterm infants. *Neonatology* **2014**, *105*, 175-181. https://doi.org/10.1159/000356782.
50. Grosfeld, J.L.; Chaet, M.; Molinari, F.; Engle, W.; Engum, S.A.; West, K.W.; Rescorla, F.J.; Scherer 3rd, L. Increased risk of necrotizing enterocolitis in premature infants with patent ductus arteriosus treated with indomethacin. *Annals of surgery* **1996**, *224*, 350. https://doi.org/10.1097/00000658-199609000-00011.

51. Halil, H.; Buyuktiryaki, M.; Atay, F.Y.; Yekta Oncel, M.; Uras, N. Reopening of the ductus arteriosus in preterm infants; Clinical aspects and subsequent consequences. *J. Neonatal-Perinat. Med.* **2018**, 1-7. https://doi.org/10.3233/NPM-17136.
52. Halliday, H.; Hirata, T.; Brady, J. Indomethacin therapy for large patent ductus arteriosus in the very low birth weight infant: results and complications. *Pediatrics* **1979**, *64*, 154-159. https://doi.org/10.1542/peds.64.2.154.
53. Hammoud, M.S.; Elsori, H.A.; Hanafi, E.A.; Shalabi, A.A.; Fouda, I.A.; Devarajan, L.V. Incidence and risk factors associated with the patency of ductus arteriosus in preterm infants with respiratory distress syndrome in Kuwait. *Saudi Med J.* **2003**, *24*, 982-985.
54. Härkin, P.; Härmä, A.; Aikio, O.; Valkama, M.; Leskinen, M.; Saarela, T.; Hallman, M. Paracetamol accelerates closure of the ductus arteriosus after premature birth: a randomized trial. *J Pediatr.* **2016**, *177*, 72-77. e72. https://doi.org/10.1016/j.jpeds.2016.04.066.
55. Härkin, P.; Marttila, R.; Pokka, T.; Saarela, T.; Hallman, M. Morbidities associated with patent ductus arteriosus in preterm infants. Nationwide cohort study. *J. Matern.-Fetal Neonatal Med.* **2018**, *31*, 2576-2583. https://doi.org/10.1080/14767058.2017.1347921.
56. Harris, C.; Zivanovic, S.; Lunt, A.; Calvert, S.; Bisquera, A.; Marlow, N.; Peacock, J.L.; Greenough, A. Lung function and respiratory outcomes in teenage boys and girls born very prematurely. *Pediatr. Pulmonol.* **2020**, *55*, 682-689. https://doi.org/10.1002/ppul.24631.
57. Harris, S.L.; More, K.; Dixon, B.; Troughton, R.; Pemberton, C.; Horwood, J.; Ellis, N.; Austin, N. Factors affecting N-terminal pro-B-type natriuretic peptide levels in preterm infants and use in determination of haemodynamic significance of patent ductus arteriosus. *Eur. J. Pediatr.* **2018**, *177*, 521-532. https://doi.org/10.1007/s00431-018-3089-y.
58. Hintz, S.R.; Kendrick, D.E.; Vohr, B.R.; Poole, W.K.; Higgins, R.D.; Network, N.N.R. Gender differences in neurodevelopmental outcomes among extremely preterm, extremely-low-birthweight infants. *Acta Paediatr.* **2006**, *95*, 1239-1248. https://doi.org/10.1080/08035250600599727.
59. Hoodbhoy, S.A.; Cutting, H.A.; Seddon, J.A.; Campbell, M.E. Cerebral and splanchnic hemodynamics after duct ligation in very low birth weight infants. *J Pediatr.* **2009**, *154*, 196-200. e192. https://doi.org/10.1016/j.jpeds.2008.07.051.
60. Hsu, J.-H.; Yang, S.-N.; Chen, H.-L.; Tseng, H.-I.; Dai, Z.-K.; Wu, J.-R. B-type natriuretic peptide predicts responses to indomethacin in premature neonates with patent ductus arteriosus. *J Pediatr.* **2010**, *157*, 79-84. https://doi.org/10.1016/j.jpeds.2009.12.045.
61. Hsu, K.-H.; Wu, T.-W.; Wu, I.-H.; Lai, M.-Y.; Hsu, S.-Y.; Huang, H.-W.; Mok, T.-Y.; Lee, C.-C.; Lien, R. Baseline cardiac output and its alterations during ibuprofen treatment for patent ductus arteriosus in preterm infants. *BMC Pediatr.* **2019**, *19*, 1-8. https://doi.org/10.1186/s12887-019-1560-1.
62. Inayat, M.; Bany-Mohammed, F.; Valencia, A.; Tay, C.; Jacinto, J.; Aranda, J.V.; Beharry, K.D. Antioxidants and biomarkers of oxidative stress in preterm infants with symptomatic patent ductus arteriosus. *American J. Perinatol.* **2015**, *32*, 895-904. https://doi.org/10.1055/s-0035-1544948.
63. Isayama, T.; Mirea, L.; Mori, R.; Kusuda, S.; Fujimura, M.; Lee, S.K.; Shah, P.S.; Japan, N.R.N.o.; Network, C.N. Patent ductus arteriosus management and outcomes in Japan and Canada: comparison of proactive and selective approaches. *American J. Perinatol.* **2015**, *32*, 1087-1094. https://doi.org/10.1055/s-0035-1548727.
64. Ito, M.; Tamura, M.; Namba, F.; Japan, N.R.N.o. Role of sex in morbidity and mortality of very premature neonates. *Pediatr. Int.* **2017**, *59*, 898-905. https://doi.org/10.1111/ped.13320.
65. Janz-Robinson, E.M.; Badawi, N.; Walker, K.; Bajuk, B.; Abdel-Latif, M.E.; Bowen, J.; Sedgley, S.; Carlisle, H.; Smith, J.; Craven, P. Neurodevelopmental outcomes of premature infants treated for patent ductus arteriosus: a population-based cohort study. *J Pediatr.* **2015**, *167*, 1025-1032. e1023. https://doi.org/10.1016/j.jpeds.2015.06.054.
66. Jennische, M.; Sedin, G. Gender differences in outcome after neonatal intensive care: speech and language skills are less influenced in boys than in girls at 6.5 years. *Acta Paediatr.* **2003**, *92*, 364-378. https://doi.org/10.1111/j.1651-2227.2003.tb00560.x.

67. Jhaveri, N.; Moon-Grady, A.; Clyman, R.I. Early surgical ligation versus a conservative approach for management of patent ductus arteriosus that fails to close after indomethacin treatment. *J Pediatr.* **2010**, *157*, 381-387. e381. https://doi.org/10.1016/j.jpeds.2010.02.062.
68. Jim, W.-T.; Chiu, N.-C.; Chen, M.-R.; Hung, H.-Y.; Kao, H.-A.; Hsu, C.-H.; Chang, J.-H. Cerebral hemodynamic change and intraventricular hemorrhage in very low birth weight infants with patent ductus arteriosus. *Ultrasound Med. Biol.* **2005**, *31*, 197-202. https://doi.org/10.1016/j.ultrasmedbio.2004.10.006.
69. Jones, H.P.; Karuri, S.; Cronin, C.M.; Ohlsson, A.; Peliowski, A.; Synnes, A.; Lee, S.K. Actuarial survival of a large Canadian cohort of preterm infants. *BMC Pediatr.* **2005**, *5*, 1-13. https://doi.org/10.1186/1471-2431-5-40.
70. Juujärvi, S.; Saarela, T.; Hallman, M.; Aikio, O. Intravenous paracetamol was associated with closure of the ductus arteriosus in extremely premature infants. *Acta Paediatr.* **2018**, *107*, 605-610. https://doi.org/10.1111/apa.14137.
71. Kahvecioglu, D.; Erdeve, O.; Akduman, H.; Ucar, T.; Alan, S.; Çakır, U.; Yıldız, D.; Atasay, B.; Arsan, S.; Atalay, S. Influence of platelet count, platelet mass index, and platelet function on the spontaneous closure of ductus arteriosus in the prematurity. *Pediatr. Neonatol* **2018**, *59*, 53-57. https://doi.org/10.1016/j.pedneo.2017.01.006.
72. Kara, M.; Bilen, M.M.; Tekgündüz, K.Ş.; Laloğlu, F.; Ceviz, N. Relation of shunt index with the patent ductus arteriosus among preterm infants under 30 weeks or 1500 g. *J. Matern.-Fetal Neonatal Med.* **2019**, 1-6. https://doi.org/10.1080/14767058.2019.1594191.
73. Karabulut, B.; Arcagök, B.C.; Simsek, A. Utility of the Platelet-to-Lymphocyte Ratio in Diagnosing and Predicting Treatment Success in Preterm Neonates with Patent Ductus Arteriosus. *Fetal Pediatr. Pathol.* **2019**, 1-10. https://doi.org/10.1080/15513815.2019.1686786.
74. Katheria, V.; Poeltler, D.; Brown, M.; Hassen, K.; Patel, D.; Rich, W.; Finer, N.; Katheria, A. Early prediction of a significant patent ductus arteriosus in infants< 32 weeks gestational age. *J. Neonatal-Perinat. Med.* **2018**, 1-7. https://doi.org/10.3233/NPM-1771.
75. Kaur, S.; Stritzke, A.; Soraisham, A.S. Does Postmenstrual Age Affect Medical Patent Ductus Arteriosus Treatment Success in Preterm Infants? *American J. Perinatol.* **2019**. *36(14)*, 1504-1509. https://doi.org/10.1055/s-0039-1678555.
76. Kazanci, E.G.; Buyuktiryaki, M.; Unsal, H.; Tayman, C. Useful platelet indices for the diagnosis and follow-up of patent ductus arteriosus. *American J. Perinatol.* **2019**, *36*, 1521-1527. https://doi.org/10.1055/s-0039-1688821.
77. Kent, A.L.; Wright, I.M.; Abdel-Latif, M.E. Mortality and adverse neurologic outcomes are greater in preterm male infants. *Pediatrics-English Edition* **2012**, *129*, 124. https://doi.org/10.1542/peds.2011-1578.
78. Kim, E.S.; Kim, E.-K.; Choi, C.W.; Kim, H.-S.; Kim, B.I.; Choi, J.-H.; Park, J.S.; Moon, K.C. Intrauterine inflammation as a risk factor for persistent ductus arteriosus patency after cyclooxygenase inhibition in extremely low birth weight infants. *J Pediatr.* **2010**, *157*, 745-750. e741. https://doi.org/10.1016/j.jpeds.2010.05.020.
79. Kim, J.S.; Shim, E.J. B-type natriuretic Peptide assay for the diagnosis and prognosis of patent ductus arteriosus in preterm infants. *Korean circulation journal* **2012**, *42*, 192-196. https://doi.org/10.4070/kcj.2012.42.3.192.
80. Kwinta, P.; Rudzinski, A.; Kruczek, P.; Kordon, Z.; Pietrzyk, J.J. Can early echocardiographic findings predict patent ductus arteriosus? *Neonatology* **2009**, *95*, 141-148. https://doi.org/10.1159/000153098
81. Laughon, M.; Bose, C.; Clark, R. Treatment strategies to prevent or close a patent ductus arteriosus in preterm infants and outcomes. *J. Perinatol.* **2007**, *27*, 164. https://doi.org/10.1038/sj.jp.7211662.
82. Lauterbach, M.D.; Raz, S.; Sander, C.J. Neonatal hypoxic risk in preterm birth infants: the influence of sex and severity of respiratory distress on cognitive recovery. *Neuropsychology* **2001**, *15*, 411. https://doi.org/10.1037/0894-4105.15.3.411.
83. Ledo, A.; Aguar, M.; Núñez-Ramiro, A.; Saénz, P.; Vento, M. Abdominal near-infrared spectroscopy detects low mesenteric perfusion early in preterm infants with hemodynamic significant ductus arteriosus. *Neonatology* **2017**, *112*, 238-245. https://doi.org/10.1159/000475933.

84. Leonhardt, A.; Isken, V.; Kühl, P.; Seyberth, H. Prolonged indomethacin treatment in preterm infants with symptomatic patent ductus arteriosus: efficacy, drug level monitoring, and patient selection. *Eur. J. Pediatr.* **1987**, *146*, 140-144. https://doi.org/10.1007/BF02343219.
85. Letshwiti, J.B.; Sirc, J.; O'Kelly, R.; Miletin, J. Serial N-terminal pro-brain natriuretic peptide measurement as a predictor of significant patent ductus arteriosus in preterm infants beyond the first week of life. *Eur J. Pediatr.***2014**, *173*, 1491-1496. https://doi.org/10.1007/s00431-014-2350-2
86. Li, D.; Rosito, G.; Slagle, T. Probiotics for the prevention of necrotizing enterocolitis in neonates: an 8-year retrospective cohort study. *J. Clin. Pharm. Ther.* **2013**, *38*, 445-449. https://doi.org/10.1111/jcpt.12084.
87. Louis, D.; Wong, C.; Ye, X.Y.; McNamara, P.J.; Jain, A. Factors associated with non-response to second course indomethacin for PDA treatment in preterm neonates. *J. Matern.-Fetal Neonatal Med.* **2018**, *31*, 1407-1411. https://doi.org/10.1080/14767058.2017.1317736.
88. Madan, J.; Fiascone, J.; Balasubramanian, V.; Griffith, J.; Hagadorn, J.I. Predictors of ductal closure and intestinal complications in very low birth weight infants treated with indomethacin. *Neonatology* **2008**, *94*, 45-51. https://doi.org/10.1159/000113058.
89. Madan, J.C.; Kendrick, D.; Hagadorn, J.I.; Frantz III, I.D.; Health, N.I.o.C.; Network, H.D.N.R. Patent ductus arteriosus therapy: impact on neonatal and 18-month outcome. *Pediatrics* **2009**, *123*, 674. https://doi.org/10.1542/peds.2007-2781.
90. Martin, C.G.; Snider, A.R.; Katz, S.M.; Peabody, J.L.; Brady, J.P. Abnormal cerebral blood flow patterns in preterm infants with a large patent ductus arteriosus. *J. Pediatr.* **1982**, *101*, 587-593. https://doi.org/10.1016/S0022-3476(82)80715-4.
91. Meinarde, L.; Hillman, M.; Rizzotti, A.; Basquiera, A.L.; Tabares, A.; Cuestas, E. C-reactive protein, platelets, and patent ductus arteriosus. *Platelets* **2016**, *27*, 821-823. https://doi.org/10.1080/09537104.2016.1203398
92. Mellander, M.; Leheup, B.; Lindstrom, D.P.; Palme, C.; Graham Jr, T.P.; Stahlman, M.T.; Cotton, R.B. Recurrence of symptomatic patent ductus arteriosus in extremely premature infants, treated with indomethacin. *J Pediatr.* **1984**, *105*, 138-143. https://doi.org/10.1016/S0022-3476(84)80380-7.
93. Mine, K.; Ohashi, A.; Tsuji, S.; Nakashima, J.; Hirabayashi, M.; Kaneko, K. B-type natriuretic peptide for assessment of haemodynamically significant patent ductus arteriosus in premature infants. *Acta Paediatr* **2013**, *102*, e347-352. https://doi.org/10.1111/apa.12273
94. Natarajan, G.; Shankaran, S.; McDonald, S.A.; Das, A.; Ehrenkranz, R.A.; Goldberg, R.N.; Stoll, B.J.; Tyson, J.E.; Higgins, R.D.; Schendel, D. Association Between Blood Spot Transforming Growth Factor-β and Patent Ductus Arteriosus in Extremely Low-Birth Weight Infants. *Pediatr. Cardiol.* **2013**, *34*, 149-154. https://doi.org/10.1007/s00246-012-0404-7.
95. Nemerofsky, S.L.; Parravicini, E.; Bateman, D.; Kleinman, C.; Polin, R.A.; Lorenz, J.M. The ductus arteriosus rarely requires treatment in infants> 1000 grams. *American J. Perinatol.* **2008**, *25*, 661-666. https://doi.org/10.1055/s-0028-1090594.
96. Neubauer, V.; Griesmaier, E.; Ralser, E.; Kiechl-Kohlendorfer, U. The effect of sex on outcome of preterm infants–a population-based survey. *Acta Paediatr.* **2012**, *101*, 906-911. https://doi.org/10.1111/j.1651-2227.2012.02709.x.
97. Nizarali, Z.; Marques, T.; Costa, C.; Barroso, R.; Cunha, M. Patent Ductus Arteriosus: Perinatal Risk Factors. *J. pediatr. neonatal biol.* **2012**. https://doi.org/10.4172/2167-0897.1000109.
98. Nuntnarumit, P.; Khositseth, A.; Thanomsingh, P. N-terminal probrain natriuretic peptide and patent ductus arteriosus in preterm infants. *J. Perinatol.* **2009**, *29*, 137. https://doi.org/10.1038/jp.2008.185.
99. Oh, S.H.; Lee, B.S.; Jung, E.; Oh, M.Y.; Do, H.-J.; Kim, E.A.-R.; Kim, K.-S. Plasma B-type natriuretic peptide cannot predict treatment response to ibuprofen in preterm infants with patent ductus arteriosus. *Sci. Rep.* **2020**, *10*, 1-7. https://doi.org/10.1038/s41598-020-61291-w.

100. Okur, N.; Tayman, C.; Büyüktiryaki, M.; Kadıoğlu Şimşek, G.; Ozer Bekmez, B.; Altuğ, N. Can lactate levels be used as a marker of patent ductus arteriosus in preterm babies? *J. Clin. Lab. Anal.* **2019**, *33*, e22664. https://doi.org/10.1002/jcla.22664.
101. Oliveira, A.; Soares, P.; Flor-de-Lima, F.; Neves, A.L.s.; Guimarães, H.l. PDA management in VLBW infants: experience of a level III NICU. *J. Pediatr. Neonatal Individ. Med.* **2016**, *5*, e050227.
102. Olsson, K.W.; Jonzon, A.; Sindelar, R. Early haemodynamically significant patent ductus arteriosus does not predict future persistence in extremely preterm infants. *Acta Paediatr.* **2019**. https://doi.org/10.1111/apa.14752.
103. Olsson, K.W.; Larsson, A.; Jonzon, A.; Sindelar, R. Exploration of potential biochemical markers for persistence of patent ductus arteriosus in preterm infants at 22–27 weeks' gestation. *Pediatr. Res.* **2018**, 1. https://doi.org/10.1038/s41390-018-0182-x.
104. Olukman, O.; Ozdemir, R.; Karadeniz, C.; Calkavur, S.; Mese, T.; Vergin, C. Is there a relationship between platelet parameters and patency of ductus arteriosus in preterm infants? *Blood Coagul. Fibrinolysis* **2017**, *28*, 8-13. https://doi.org/10.1097/MBC.0000000000000520.
105. O'Rourke, D.J.; El-Khuffash, A.; Moody, C.; Walsh, K.; Molloy, E.J. Patent ductus arteriosus evaluation by serial echocardiography in preterm infants. *Acta Paediatr* **2008**, *97*, 574-578. https://doi.org/10.1111/j.1651-2227.2008.00745.x
106. Patole, S.K.; Kumaran, V.; Travadi, J.N.; Brooks, J.M.; Doherty, D.A. Does patent ductus arteriosus affect feed tolerance in preterm neonates? *Arch Dis Child Fetal Neonatal Ed* **2007**, *92*, F53-55. https://doi.org/10.1136/adc.2006.093708.
107. Peacock, J.L.; Marston, L.; Marlow, N.; Calvert, S.A.; Greenough, A. Neonatal and infant outcome in boys and girls born very prematurely. *Pediatr. Res.* **2012**, *71*, 305. https://doi.org/10.1038/pr.2011.50.
108. Pees, C.; Walch, E.; Obladen, M.; Koehne, P. Echocardiography predicts closure of patent ductus arteriosus in response to ibuprofen in infants less than 28 week gestational age. *Early Hum Dev* **2010**, *86*, 503-508. https://doi.org/10.1016/j.earlhumdev.2010.06.012.
109. Pereira, S.S.; Kempley, S.T.; Shah, D.K.; Morris, J.K.; Sinha, A.K. Early echocardiography does not predict subsequent treatment of symptomatic patent ductus arteriosus in extremely preterm infants. *Acta Paediatr.* **2018**, *107*, 1909-1916. https://doi.org/10.1111/apa.14361.
110. Polat, T.B.; Celik, I.H.; Erdeve, O. Early predictive echocardiographic features of hemodynamically significant patent ductus arteriosus in preterm VLBW infants. *Pediatr. Int.* **2016**, *58*, 589-594. https://doi.org/10.1111/ped.12915.
111. Pourarian, S.; Farahbakhsh, N.; Sharma, D.; Cheriki, S.; Bijanzadeh, F. Prevalence and risk factors associated with the patency of ductus arteriosus in premature neonates: a prospective observational study from Iran. *J. Matern.-Fetal Neonatal Med.* **2017**, *30*, 1460-1464. https://doi.org/10.1080/14767058.2016.1219991.
112. Reller, M.D.; Lorenz, J.M.; Kotagal, U.R.; Meyer, R.A.; Kaplan, S. Hemodynamically significant PDA: an echocardiographic and clinical assessment of incidence, natural history, and outcome in very low birth weight infants maintained in negative fluid balance. *Pediatr. Cardiol* **1985**, *6*, 17-23. https://doi.org/10.1007/BF02265403.
113. Rodriguez-Blanco, S.; Oulego-Erroz, I.; Gautreaux-Minaya, S.; Perez-Muñuzuri, A.; Couce-Pico, M.L. Early NT-proBNP levels as a screening tool for the detection of hemodynamically significant patent ductus arteriosus during the first week of life in very low birth weight infants. *J. Perinatol.* **2018**, *38*, 881. https://doi.org/10.1038/s41372-018-0123-x.
114. Romagnoli, V.; Pedini, A.; Santoni, M.; Scutti, G.; Colaneri, M.; Pozzi, M.; Cogo, P.E.; Carnielli, V.P. Patent ductus arteriosus in preterm infants born before 30 weeks' gestation: high rate of spontaneous closure after hospital discharge. *Cardiol. Young* **2018**, *28*, 995-1000. https://doi.org/10.1017/S1047951118000641.
115. Rooney, S.R.; Shelton, E.L.; Aka, I.; Shaffer, C.M.; Clyman, R.I.; Dagle, J.M.; Ryckman, K.; Lewis, T.R.; Reese, J.; Van Driest, S.L. CYP2C9* 2 is associated with indomethacin treatment failure for patent ductus arteriosus. *Pharmacogenomics* **2019**, *20*, 939-946. https://doi.org/10.2217/pgs-2019-0079.

116. Sadeck, L.S.; Leone, C.R.; Procianoy, R.S.; Guinsburg, R.; Marba, S.; Martinez, F.E.; Rugolo, L.M.; Moreira, M.E.L.; Fiori, R.M.; Ferrari, L.L. Effects of therapeutic approach on the neonatal evolution of very low birth weight infants with patent ductus arteriosus. *J. Pediatr* **2014**, *90*, 616-623. https://doi.org/10.1016/j.jped.2014.04.010.
117. Sallmon, H.; Weber, S.C.; Dirks, J.; Schiffer, T.; Klippstein, T.; Stein, A.; Felderhoff-Müser, U.; Metze, B.; Hansmann, G.; Bührer, C. Association between platelet counts before and during pharmacological therapy for patent ductus arteriosus and treatment failure in preterm infants. *Front. Pediatr.* **2018**, *6*, 41. https://doi.org/10.3389/fped.2018.00041.
118. Sallmon, H.; Weber, S.C.; Huning, B.; Stein, A.; Horn, P.A.; Metze, B.C.; Dame, C.; Buhrer, C.; Felderhoff-Muser, U.; Hansmann, G.; et al. Thrombocytopenia in the first 24 hours after birth and incidence of patent ductus arteriosus. *Pediatrics* **2012**, *130*, e623-630. https://doi.org/10.1542/peds.2012-0499.
119. Sanjeev, S.; Pettersen, M.; Lua, J.; Thomas, R.; Shankaran, S.; L'Ecuyer, T. Role of plasma B-type natriuretic peptide in screening for hemodynamically significant patent ductus arteriosus in preterm neonates. *J. Perinatol.* **2005**, *25*, 709-713. https://doi.org/10.1038/sj.jp.7211383.
120. Schwarz, C.E.; Preusche, A.; Wolf, M.; Poets, C.F.; Franz, A.R. Prospective observational study on assessing the hemodynamic relevance of patent ductus arteriosus with frequency domain near-infrared spectroscopy. *BMC Pediatr.* **2018**, *18*, 66. https://doi.org/10.1186/s12887-018-1054-6.
121. Sellmer, A.; Bjerre, J.V.; Schmidt, M.R.; McNamara, P.J.; Hjortdal, V.E.; Høst, B.; Bech, B.H.; Henriksen, T.B. Morbidity and mortality in preterm neonates with patent ductus arteriosus on day 3. *Arch. Dis. Child. Fetal Neonatal Ed.* **2013**, *98*, F505-F510. https://doi.org/10.1136/archdischild-2013-303816.
122. Semberova, J.; Sirc, J.; Miletin, J.; Kucera, J.; Berka, I.; Sebkova, S.; O'Sullivan, S.; Franklin, O.; Stranak, Z. Spontaneous closure of patent ductus arteriosus in infants≤ 1500 g. *Pediatrics* **2017**, *140*, e20164258. https://doi.org/10.1542/peds.2016-4258.
123. Seon, H.-S.; Lee, J.-B.; Kim, I.-U.; Kim, S.-H.; Lee, J.-H.; Kim, D.-H.; Kim, H.-S. Association with ductus arteriosus closure by ibuprofen and intrauterine inflammation in very low birth weight infants. *Korean J. Perinatol.* **2013**, *24*, 158-167. https://doi.org/10.14734/kjp.2013.24.3.158.
124. Shah, N.A.; Hills, N.K.; Waleh, N.; McCurnin, D.; Seidner, S.; Chemtob, S.; Clyman, R. Relationship between circulating platelet counts and ductus arteriosus patency after indomethacin treatment. *J. Pediatr.***2011**, *158*, 919-923 e911-912. https://doi.org/10.1016/j.jpeds.2010.11.018.
125. Skiöld, B.; Alexandrou, G.; Padilla, N.; Blennow, M.; Vollmer, B.; Ådén, U. Sex differences in outcome and associations with neonatal brain morphology in extremely preterm children. *J Pediatr.* **2014**, *164*, 1012-1018. https://doi.org/10.1016/j.jpeds.2013.12.051.
126. Stark, M.J.; Hodyl, N.A.; Wright, I.M.; Clifton, V. The influence of sex and antenatal betamethasone exposure on vasoconstrictors and the preterm microvasculature. *J. Matern.-Fetal Neonatal Med.* **2011**, *24*, 1215-1220. https://doi.org/10.3109/14767058.2011.569618.
127. Steiner, M.; Salzer-Muhar, U.; Swoboda, V.; Unterasinger, L.; Baumgartner, S.; Waldhoer, T.; Langgartner, M.; Klebermass-Schrehof, K.; Berger, A. Preterm infants who later require duct ligation show different vital signs and pH in early postnatal life. *Acta Paediatr.* **2015**, *104*, e7-e13. https://doi.org/10.1111/apa.12814.
128. Stevenson, D.K.; Verter, J.; Fanaroff, A.A.; Oh, W.; Ehrenkranz, R.A.; Shankaran, S.; Donovan, E.F.; Wright, L.L.; Lemons, J.A.; Tyson, J.E. Sex differences in outcomes of very low birthweight infants: the newborn male disadvantage. *Arch. Dis. Child. Fetal Neonatal Ed.* **2000**, *83*, F182-F185. https://doi.org/10.1136/fn.83.3.F182.
129. Strauss, T.; Pessach, I.; Jacoby, E.; Schushan-Eisen, I.; Mazkereth, R.; Kuint, J. Carina angle measurements for diagnosis of patent ductus arteriosus in preterm infants. *Neonatology* **2011**, *99*, 224-230. https://doi.org/10.1159/000315862.
130. Tauber, K.; Granina, E.; Doyle, R.; Munshi, U. Gestational and postnatal age influence B-type natriuretic peptide level used in diagnosis of a hemodynamically significant patent ductus arteriosus in preterm infants. *J. Clin. Neonatol.* **2016**, *5*, 143-149. https://doi.org/10.4103/2249-4847.191241
131. Terek, D.; Yalaz, M.; Ulger, Z.; Koroglu, O.A.; Kultursay, N. Medical closure of patent ductus arteriosus does not reduce mortality and development of bronchopulmonary dysplasia in preterm infants. *J. Res. Med. Sci.* **2014**, *19*, 1074. https://doi.org/10.1038/nm0913-1074.

132. Tioseco, J.A.; Aly, H.; Essers, J.; Patel, K.; El-Mohandes, A.A. Male sex and intraventricular hemorrhage. *Pediatr. Crit. Care Med.* **2006**, *7*, 40-44. https://doi.org/10.1097/01.PCC.0000192341.67078.61.
133. Uchiyama, A.; Nagasawa, H.; Yamamoto, Y.; Tatebayashi, K.; Suzuki, H.; Yamada, K.; Arai, M.; Kohno, Y. Clinical aspects of very-low-birthweight infants showing reopening of ductus arteriosus. *Pediatr. Int.* **2011**, *53*, 322-327. https://doi.org/10.1111/j.1442-200X.2010.03251.x.
134. Vaidya, R.; Knee, A.; Paris, Y.; Singh, R. Predictors of successful patent ductus arteriosus closure with acetaminophen in preterm infants. *J. Perinatol.* **2021**, *41*, 998-1006. https://doi.org/10.1038/s41372-020-00803-y.
135. Van de Bor, M.; Verloove-Vanhorick, S.P.; Brand, R.; Ruys, J.H. Patent ductus arteriosus in a cohort of 1338 preterm infants: a collaborative study. *Paediatr. Perinat. Epidemiol.* **1988**, *2*, 328-336. https://doi.org/10.1111/j.1365-3016.1988.tb00227.x.
136. Velazquez, D.M.; Reidy, K.J.; Sharma, M.; Kim, M.; Vega, M.; Havranek, T. The effect of hemodynamically significant patent ductus arteriosus on acute kidney injury and systemic hypertension in extremely low gestational age newborns. *J. Matern.-Fetal Neonatal Med.* **2018**, 1-6. https://doi.org/10.1080/14767058.2018.1460349.
137. Vieux, R.; Desandes, R.; Boubred, F.; Semama, D.; Guillemin, F.; Buchweiller, M.-C.; Fresson, J.; Hascoet, J.-M. Ibuprofen in very preterm infants impairs renal function for the first month of life. *Pediatr. Nephrol.* **2010**, *25*, 267-274. https://doi.org/10.1007/s00467-009-1349-9.
138. Visconti, L.F.; Morhy, S.S.; Deutsch, A.D.; Tavares, G.M.; Wilberg, T.J.; Rossi Fde, S. Clinical and echocardiographic characteristics associated with the evolution of the ductus arteriosus in the neonate with birth weight lower than 1,500g. *Einstein (Sao Paulo)* **2013**, *11*, 317-323. https://doi.org/10.1590/S1679-45082013000300010.
139 Weiss, H.; Cooper, B.; Brook, M.; Schlueter, M.; Clyman, R. Factors determining reopening of the ductus arteriosus after successful clinical closure with indomethacin. *J Pediatr.* **1995**, *127*, 466-471. https://doi.org/10.1016/S0022-3476(95)70084-6.
140. Weisz, D.E.; Mirea, L.; Rosenberg, E.; Jang, M.; Ly, L.; Church, P.T.; Kelly, E.; Kim, S.J.; Jain, A.; McNamara, P.J. Association of patent ductus arteriosus ligation with death or neurodevelopmental impairment among extremely preterm infants. *JAMA Pediatr.* **2017**, *171*, 443-449. https://doi.org/10.1001/jamapediatrics.2016.5143.
141. Yang, C.-Z.; Lee, J. Factors affecting successful closure of hemodynamically significant patent ductus arteriosus with indomethacin in extremely low birth weight infants. *World J. Clin. Pediatr* **2008**, *4*, 91-96. https://doi.org/10.1007/s12519-008-0017-7.
142. Yoon, M.J.; Yoon, H.S.; Chung, S.H.; Han, M.Y.; Bae, C.W. The factors associated with the efficacy of indomethacin treatment in premature infants with patent ductus arteriosus. *Korean J. Pediatr.* **2007**, *50*, 531-535. https://doi.org/10.3345/kjp.2007.50.6.531.
143. Yum, S.K.; Moon, C.-J.; Youn, Y.-A.; Lee, J.Y.; Sung, I.K. Echocardiographic assessment of patent ductus arteriosus in very low birthweight infants over time: prospective observational study. *J. Matern.-Fetal Neonatal Med.* **2018**, *31*, 164-172. https://doi.org/10.1080/14767058.2016.1278207.
144. Zanardo, V.; Vedovato, S.; Chiozza, L.; Faggian, D.; Favaro, F.; Trevisanuto, D. Pharmacological closure of patent ductus arteriosus: effects on pulse pressure and on endothelin-1 and vasopressin excretion. *American J. Perinatol.* **2008**, *25*, 353-358. https://doi.org/10.1055/s-2008-1078763.
145. Zisk, J.L.; Genen, L.H.; Kirkby, S.; Webb, D.; Greenspan, J.; Dysart, K. Do premature female infants really do better than their male counterparts? *American J. Perinatol.* **2011**, *28*, 241-246. https://doi.org/10.1055/s-0030-1268239.
146. Zozaya, C.; Avila-Alvarez, A.; Arruza, L.; Rodrigo, F.G.-M.; Fernandez-Perez, C.; Castro, A.; Cuesta, M.T.; Vacas, B.; Couce, M.L.; Torres, M.V. The effect of morbidity and sex on postnatal growth of very preterm infants: a multicenter cohort study. *Neonatology* **2019**, *115*, 348-354. https://doi.org/10.1159/000497221.

Table S3. Meta-regression of continuous covariates.

Meta-analysis	Covariate	K	*Coefficient*	95% CI		P	R^2 analog
				Lower limit	Upper limit		
**Any PDA vs. closed DA**	GA total cohort (weeks)	80	0.002	-0.012	0.020	0.871	0.00
	Median year of cohort	72	-0.002	-0.006	0.002	0.332	0.00
**hsPDA vs.(small PDA + closed DA)**	GA total cohort (weeks)	36	0.014	-0.020	0.049	0.408	0.00
	Median year of cohort	34	0.000	-0.010	0.010	0.998	0.00
**hsPDA vs small PDA**	GA total cohort (weeks)	28	-0.009	-0.062	0.043	0.723	0.00
	Median year of cohort	23	0.009	0.001	0.0160	0.884	0.00
**Responders vs non responders**	GA total cohort (weeks)	45	-0.016	-0.039	0.007	0.169	0.00
	Median year of cohort	41	0.003	-0.005	0.005	0.965	0.00

GA: gestational age; hs: hemodynamically significant; PDA: patent ductus arteriosus

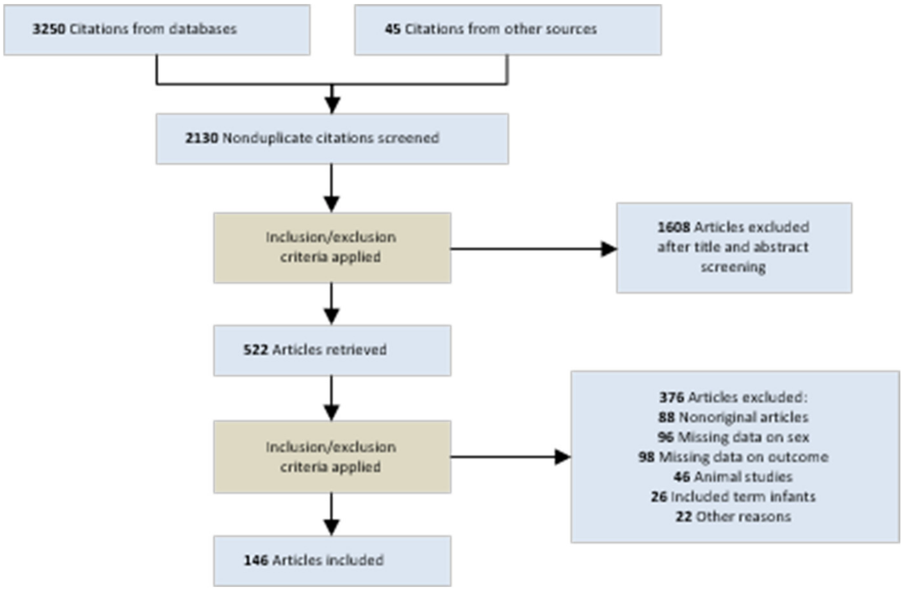

Figure S1. Flow diagram of the systematic search.

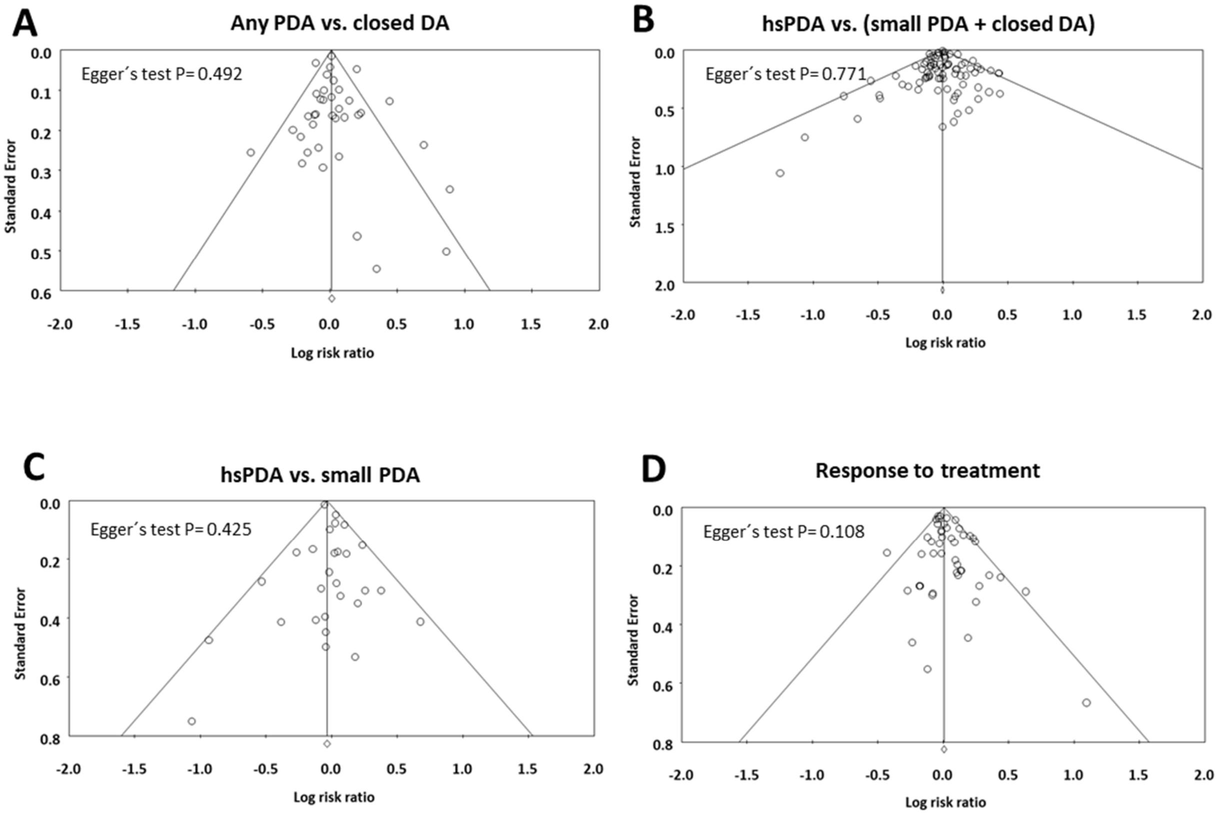

Figure S2. Funnel plot for publication bias analysis for the studies included in the different meta-analyses.

hs: hemodynamically significant. PDA: patent ductus arteriosus
